# Unique challenges in the Turkish workforce: examining the impact of job demands and resources on work-related outcomes of blue- and white-collar workers

**DOI:** 10.3389/fpsyg.2025.1588266

**Published:** 2025-07-02

**Authors:** Ümit Deniz İlhan, Burcu Kümbül Güler, Dilara Turgut, Cem Duran

**Affiliations:** ^1^Department of Business Administration, Beykoz University, İstanbul, Türkiye; ^2^Department of Psychology, Izmir Kâtip Çelebi University, İzmir, Türkiye; ^3^Department of Management Information Systems, Istinye University, İstanbul, Türkiye

**Keywords:** job demands-resources theory, work-related outcomes, blue-collar workers, white-collar workers, Turkish workforce, strategic management, human resources management

## Abstract

**Introduction:**

An increasing body of research has explored the impact of job demands and resources on employee wellbeing and performance; however, empirical evidence remains limited regarding how these factors operate in non-Western contexts. Addressing this gap, the present study examines blue-collar (70.7%) and white-collar (29.3%) workers in Türkiye, investigating the effects of job demands (quantitative, cognitive, emotional) and job resources (leader support, co-worker support, trust) on emotional exhaustion, job satisfaction, organizational commitment, and intention to leave.

**Method:**

A total of 563 employees from three large production companies in Türkiye voluntarily participated in this study. Data were collected using the Turkish version of the Copenhagen Psychosocial Questionnaire (COPSOQ-III) for all constructs, except for intention to leave, which was assessed using the Michigan Organizational Assessment Questionnaire (MOAQ). The proposed relationships were analyzed through multi-group structural equation modeling (MG-SEM).

**Results:**

Job demands significantly increased emotional exhaustion in both blue- and white-collar workers. However, while emotional exhaustion did not diminish organizational commitment among blue-collar workers, job satisfaction emerged as a key determinant in reducing their turnover intentions. In contrast, for white-collar workers, emotional exhaustion weakened organizational commitment, ultimately leading to higher turnover intentions. Moreover, job resources enhanced job satisfaction and indirectly reduced turnover intentions for blue-collar workers, whereas these resources did not significantly predict job satisfaction among white-collar workers.

**Conclusion:**

These findings underscore the importance of tailoring workplace strategies to different occupational groups in a non-Western setting. Strengthening job resources—particularly trust, leader support, and co-worker support—can enhance job satisfaction and reduce turnover risk among blue-collar workers. Conversely, for white-collar workers, mitigating job demands and managing emotional exhaustion are crucial for sustaining organizational commitment. This study contributes to the cross-cultural understanding of job demands and resources, highlighting their differential impact on blue- and white-collar workers in Türkiye.

## 1 Introduction

The modern labor market is becoming increasingly unstable due to dynamics such as economic uncertainties, job insecurity, and rising workloads. These challenges not only threaten job stability but also negatively impact employee wellbeing, highlighting the necessity of sustainable and healthy work environments (World Economic Forum, 2023). While job-related stress is a globally prevalent issue, it is particularly pronounced in developing economies, where factors such as high inflation, workforce restructuring, and limited career mobility exacerbate financial pressure, further deepening job insecurity among employees (Kortum et al., 2010; Kortum and Leka, 2014).

As a developing country, Türkiye has experienced multiple economic crises, including the 2001 financial crisis and the 2008 global recession, both of which have adversely affected economic growth, increased unemployment rates, and negatively influenced productivity and wage levels (Koseoglu et al., 2021; Toksoz, 2008). These global and domestic economic challenges have profoundly shaped the labor market, which is characterized by demanding working conditions, widespread job dissatisfaction, and high turnover rates. Empirical data indicate that Turkish employees work extended hours, with an average of 43.9 h per week, and ~28% exceeding 49 h (ILO, 2025). Prolonged working hours are strongly correlated with increased occupational stress; for instance, research suggests that nearly half (47%) of employees are on the verge of quiet quitting, while 24% have already disengaged from their work (Youthall, 2022). In addition to excessive working hours, poor working conditions further exacerbate employee dissatisfaction, in some cases exerting a more detrimental impact on psychological wellbeing than unemployment itself. The prevalence of toxic work environments and chronic dissatisfaction significantly contribute to mental health challenges among employees (Ugur, 2023). This instability is further reflected in employee turnover rates, with findings from PERYÖN (2018) indicating an annual turnover rate of 24.45%, which has escalated to 34% by the end of the first quarter of 2024. Additionally, 63% of employers report significant challenges in maintaining employee engagement, while 77% of workers have left their positions in pursuit of better salaries and improved working conditions (Randstad, 2023). Beyond retention difficulties, job stability remains a critical issue within the Turkish labor market, primarily due to insufficient job security, which is a major contributor to heightened anxiety levels among employees (Koseoglu et al., 2021). Given these interrelated challenges, a comprehensive approach to improving working conditions, fostering job security, and enhancing employee engagement is essential to ensuring long-term organizational sustainability.

To analyze these dynamics, this study adopts the Job Demands-Resources (JD-R) theory, which distinguishes between job demands—factors that deplete energy and contribute to burnout—and job resources, that enhance motivation and strengthen organizational commitment (Bakker and Demerouti, 2017). While extensive research has been conducted on the JD-R theory, the majority of studies have been situated within Western contexts, thereby creating a significant gap in understanding how these dynamics manifest in non-Western work environments (Kim and Wang, 2018; Tomczak and Kulikowski, 2023). Türkiye presents a distinctive case, as the work stressors, coping mechanisms, job attitudes, and organizational expectations of blue- and white-collar workers differ considerably (Ersoy et al., 2011; Saraç et al., 2017; Camgoz et al., 2024). Investigating these distinctions within the Turkish labor market contributes to a broader understanding of the JD-R theory's applicability in diverse socio-economic and cultural contexts, offering valuable insights for both scholars and practitioners in the field of organizational behavior and human resource management.

Furthermore, the majority of existing studies have primarily focused on a single occupational group, predominantly white-collar workers, thereby overlooking the distinct work stressors and resources that influence blue-collar workers (Schreurs et al., 2011). Given that a substantial segment of Türkiye's labor market comprises blue-collar workers, particularly within industries such as manufacturing and production, a comparative analysis of these two workforce segments is essential. Such an approach would facilitate a more comprehensive understanding of job-related stressors, coping mechanisms, and employee retention dynamics, ultimately contributing to the development of more inclusive organizational strategies tailored to diverse occupational groups.

By addressing a significant gap in JD-R theory within non-Western contexts, this study offers a multi-dimensional contribution to the theoretical refinement of the JD-R framework. First, it advances the model by incorporating occupational segmentation into its structure, empirically demonstrating that the motivational and health-impairment pathways operate differently for blue- and white-collar workers. Second, the study introduces sequential mediation mechanisms—specifically, the distinct chains through which job demands and resources influence turnover intentions via emotional exhaustion, job satisfaction, and organizational commitment—thus deepening the understanding of dynamic processes embedded in the JD-R theory. Third, it contextualizes these pathways within Türkiye's high power distance and collectivist work culture, showing that interpersonal job resources (e.g., supervisor support, co-worker support, trust) hold differential salience across occupational groups. In doing so, the study not only validates the JD-R framework in a culturally distinct labor market but also challenges its generalized assumptions by revealing how socio-cultural factors shape the perceived value and function of job demands and resources. These theoretical insights contribute meaningfully to both occupational health psychology and international human resource management, offering a more context-sensitive and stratified application of the JD-R theory.

## 2 Model and hypothesis development

**Figure 1:**
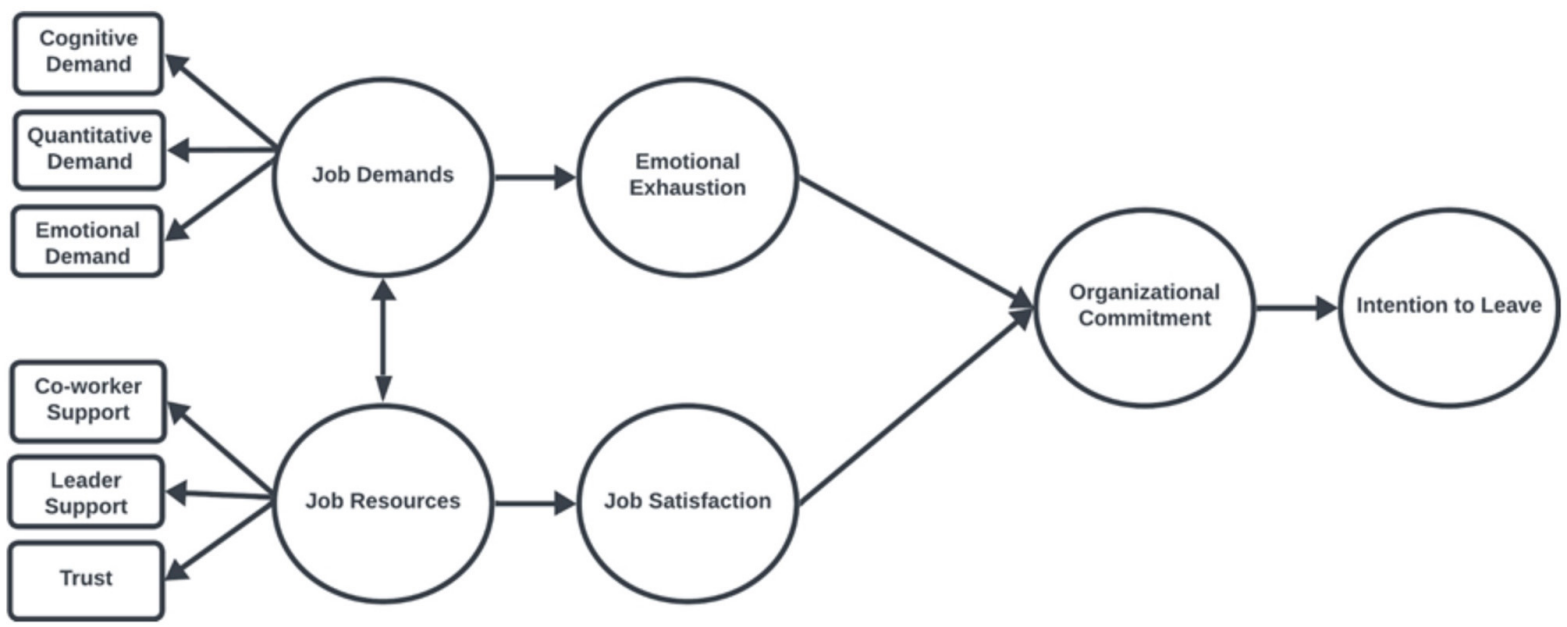
Theoretical model.

The proposed model, grounded in the JD-R theory (Bakker and Demerouti, 2006), is illustrated in Figure 1. Within this framework, job demands—quantitative, cognitive, and emotional demands—contribute to emotional exhaustion, which subsequently weakens organizational commitment and increases the intention to leave. Conversely, job resources, including co-worker support, leader support, and trust, serve as protective factors that enhance job satisfaction, thereby strengthening organizational commitment and reducing turnover intentions.

### 2.1 Job demands-resources (JD-R) theory

The JD-R theory provides a comprehensive framework for understanding the impact of job characteristics on employee wellbeing and performance by categorizing them into job demands and job resources. While job demands require sustained effort, potentially leading to strain and burnout, job resources enhance motivation, support personal development, and mitigate the adverse effects of excessive demands. This dual mechanism operates through two distinct pathways: the health impairment process, where excessive job demands drain employees' energy, leading to emotional exhaustion and increased turnover intention, and the motivational process, where sufficient job resources enhance job satisfaction and organizational commitment, ultimately reducing turnover (Bakker et al., 2007; Demerouti et al., 2001; Schaufeli and Bakker, 2004).

This study examines three key job demands—quantitative, cognitive, and emotional—as they are particularly relevant in Türkiye's current labor market, which is characterized by high inflation, economic volatility, poverty, low wages, and a growing refugee influx (Büyükgöze-Kavas and Autin, 2019; Çarkit, 2024). These macroeconomic and socio-political factors exacerbate existing job demands, intensifying workload expectations (quantitative demand), increasing cognitive burdens due to the shift toward knowledge-based work (cognitive demand), and amplifying emotional strain resulting from heightened job insecurity (emotional demand) (Schaufeli and Taris, 2014). Given the ongoing economic instability and evolving labor market conditions in Türkiye, accurately measuring these job demands is crucial for understanding their impact on employees and developing context-specific strategies to support workforce wellbeing and productivity.

In terms of job resources, this study focuses on three interpersonal variables: leader support, co-worker support, and trust. Although the JD-R framework allows for a broader range of resources—including autonomy, career advancement opportunities, and role clarity—these were intentionally excluded to maintain theoretical focus and prevent model overcomplexity. In particular, autonomy and career development opportunities often activate different motivational mechanisms related to intrinsic satisfaction (Deci and Ryan, 2000; Van den Broeck et al., 2010), which fall outside the primary scope of this study.

The selection of leader support, co-worker support, and trust is also theoretically and culturally grounded. Türkiye's workplace environment is shaped by high power distance and collectivism (Fikret-Pasa et al., 2001; Hofstede, 2001), where trust in supervisors, support from peers, and respect for authority significantly influence how employees perceive and cope with job demands (Berkman and Özen, 2008; Ersoy et al., 2011). In such contexts, informal interpersonal dynamics—such as loyalty to leaders or harmony among team members—often compensate for the lack of formal organizational mechanisms. These dynamics are especially salient for blue-collar workers, who typically rely on strong workplace relationships to navigate routine pressures and achieve psychological safety (Aycan, 2006; Pellegrini and Scandura, 2008). Therefore, prioritizing interpersonal job resources provides a culturally congruent and empirically grounded framework for explaining variation in employee outcomes across occupational groups in the Turkish context.

### 2.2 Work-related outcomes

Ensuring employee wellbeing while maintaining organizational stability represents one of the most pressing challenges in today's workplace. In this context, work-related outcomes play a crucial role in shaping long-term employee commitment and influencing turnover intentions. Drawing on the JD-R theory, this study examines four key workplace outcomes: emotional exhaustion, job satisfaction, organizational commitment, and intention to leave.

Emotional exhaustion, defined as a state of chronic fatigue and diminished motivation resulting from prolonged job stress (Maslach and Jackson, 1981), is a core component of burnout that significantly undermines employees' organizational attachment (Demerouti et al., 2003; Schaufeli and Bakker, 2004). Elevated job demands, manifested in excessive workloads, cognitively complex tasks, and emotionally taxing interactions, heighten the likelihood of experiencing emotional exhaustion. Over time, persistent exposure to these stressors leads to a decline in organizational commitment and an increased likelihood of turnover intentions (Prajogo, 2019). In light of this, we hypothesize that job demands positively influence emotional exhaustion (H_1_).

Conversely, job resources serve as protective factors, enhancing employees' work experience by fostering job satisfaction, which is conceptualized as a positive emotional response reflecting the degree to which employees feel fulfilled and valued in their roles (Locke, 1969). Access to supportive resources, including effective leadership, strong co-worker support, and trust-based workplace relationships, has been shown to enhance job satisfaction (Westover and Taylor, 2010). This positive affective state not only mitigates the detrimental effects of high job demands but also reinforces organizational commitment, thereby reducing turnover intentions. Accordingly, we hypothesize that job resources positively influence job satisfaction (H_2_).

Organizational commitment, defined as an employee's emotional attachment and loyalty to their organization (Allen and Meyer, 1991), plays a mediating role in the relationship between work experiences and turnover intentions. Employees with high commitment tend to remain engaged and are less likely to leave, whereas those suffering from emotional exhaustion often become disengaged and detached (Meyer et al., 1993). Similarly, high job satisfaction strengthens commitment, as employees who find their work meaningful and rewarding are more inclined to stay within the organization (Rombaut and Guerry, 2021). Therefore, we propose that organizational commitment mediates the relationship between job satisfaction and turnover intentions (H_3_) and the relationship between emotional exhaustion and turnover intentions (H_4_).

Intention to leave refers to an employee's deliberate consideration of leaving their job (Shenoy and Sharma, 2022) and is widely recognized as a strong predictor of actual turnover. This outcome is particularly salient in high-stress environments characterized by low job satisfaction and diminished organizational commitment (Hulin, 1991; Mitchell et al., 2001). Consistent with this, job demands that contribute to emotional exhaustion weaken organizational commitment and prompt employees to seek alternative employment opportunities (Kim et al., 2017). A meta-analysis conducted in Türkiye by Pozanti et al. (2022) further supports the robust relationship between burnout and intention to leave across various industrial sectors. Additionally, a recent meta-analysis of public sector employees across multiple countries found that exhaustion is a strong predictor of intention to leave (Hur and Abner, 2024). Based on these findings, we hypothesize that the relationship between job demands and turnover intentions is sequentially mediated by emotional exhaustion and organizational commitment (H_5_). In contrast, job resources facilitate a motivational process that enhances organizational commitment and reduces turnover intentions. Employees who benefit from social support, effective leadership, and a trust-based work environment tend to experience higher job satisfaction, which in turn strengthens organizational commitment (Bakker and Demerouti, 2017). This long-term attachment reduces employees' intention to leave. Thus, we hypothesize that the relationship between job resources and turnover intentions is sequentially mediated by job satisfaction and organizational commitment (H_6_).

The proposed sequential mediation mechanisms in H_5_ and H_6_ are grounded in empirical research supporting the dual-process model of the JD-R theory (Bakker and Demerouti, 2017). Prior studies have demonstrated that job demands can indirectly lead to turnover intentions through emotional exhaustion and diminished organizational commitment (Jourdain and Chênevert, 2010; Babakus et al., 2008), while job resources can enhance job satisfaction and commitment, thereby reducing turnover (Palvimo et al., 2023; Russell et al., 2020). These studies validate the proposed mediational logic by establishing empirical pathways that mirror the theoretical propositions outlined in this research.

### 2.3 Blue- and white-collar work

Work performed by white-collar workers, characterized by skill-intensive, non-repetitive, and intellectually demanding tasks, along with higher pay and benefits, is often viewed as the antithesis of blue-collar work, which is largely routine and repetitive (Halle, 1984; Yavas, 2021). White-collar roles involve complex tasks requiring high variability, frequent interactions with diverse individuals, and advanced problem-solving abilities, necessitating strategic thinking and cross-organizational engagement. In contrast, blue-collar work is typically structured and standardized, focusing on manual or technical tasks that demand less cognitive problem-solving and strategic decision-making (Morgeson and Humphrey, 2006). Given their physically demanding nature, blue-collar careers are often perceived as having limited upward mobility, lacking the linear progression paths that are more common among white-collar workers (Hennequin, 2007).

White-collar workers face substantial cognitive, organizational, and emotional job demands. In Türkiye, these employees frequently struggle with excessive workloads, ambiguous job roles, and expectations to remain available beyond official working hours (Anxo and Karlsson, 2019). Such working conditions contribute to mental strain, work-life imbalance, and emotional exhaustion. Moreover, job insecurity is a growing concern, as rising unemployment rates and stagnating wages increase stress and uncertainty among professionals (Yavas, 2021). In contrast, blue-collar workers primarily experience physical and economic job demands, often engaging in repetitive, high-intensity physical labor under hazardous conditions (Çimrin et al., 2023). Job precarity remains a critical issue due to subcontracting, seasonal employment fluctuations, and limited career advancement opportunities (Sahin, 2021). These factors contribute to both physical and psychological strain, negatively impacting job satisfaction and long-term engagement. Empirical evidence suggests that overall job demands tend to be higher for white-collar workers than for blue-collar workers, particularly in relation to cognitive and organizational pressures. This challenges the prevailing assumption that physically demanding jobs inherently result in higher work-related stress compared to mentally intensive roles (Demiral et al., 2023).

Despite these challenges, both occupational groups rely on different job resources to maintain motivation and job satisfaction. Research by Iverson and Roy (1994) demonstrates that blue- and white-collar workers differ in their reliance on job resources when forming organizational commitment. Blue-collar workers tend to prioritize extrinsic job resources, such as fair wages, job security, and strong workplace relationships (Pennings, 1970). Additionally, peer relationships and teamwork serve as crucial non-monetary job resources, offering social recognition and emotional support in the workplace (Hennequin, 2007). White-collar workers, by contrast, often emphasize intrinsic job resources, including career development opportunities, intellectual engagement, and meaningful work (Schreuder et al., 2008). Moreover, the ability to balance work and personal life is particularly essential for white-collar workers, as blurred work-life boundaries contribute to additional stress and reduced wellbeing.

Given these dynamics, we hypothesize that job demands and job resources exert distinct yet significant influences on work-related outcomes across occupational groups (H_7_).

## 3 Materials and methods

### 3.1 Participants and procedure

The data for this study were collected from a total of 563 full-time employees, comprising both blue- and white-collar workers aged 19–56 years (*M* = 35.58, *SD* = 7.26), employed at three large production companies in Türkiye. The sample distribution included 70.7% blue-collar and 29.3% white-collar workers. The sociodemographic characteristics of the participants are detailed in Table 1 (blue-collar workers) and Table 2 (white-collar workers).

**Table 1:** Sociodemographic characteristics of blue-collar workers.

**Characteristic**		***n***	**%**
Gender	Women	57	14.3
	Men	340	85.4
	Not specified	1	0.3
Age	Min = 19	35.78	
	Max = 57		
Education	Primary	16	4.0
	Secondary	56	14.1
	High school	274	68.8
	College	39	9.8
	University	13	3.3
Marital status	Single	272	68.3
	Married	126	31.7
Shift work	Yes	346	86.9
	No	52	13.1
Tenure	0–1 year	47	11.8
	<1–3 years	36	9.0
	<3–5 years	55	13.8
	<5–10 years	108	27.1
	<10–15 years	72	18.1
	<15 years	80	20.1
Contract type	Permanent/Unionized	286	71.9%
	Temporary	112	28.1%

**Table 2:** Sociodemographic characteristics of white-collar workers.

**Characteristic**		***n***	**%**
Gender	Women	49	29.7
	Men	96	58.2
	Not specified	20	12.1
Age	Min = 22	35.11	
	Max = 56		
Education	Primary	1	0.6
	High school	25	15.2
	College	29	17.6
	University	72	43.6
	Master's	17	10.3
	PhD	1	0.6
	Not specified	20	12.1
Marital status	Single	96	58.2
	Married	45	27.3
Shift work	Yes	28	17.0
	No	120	72.7
	Not specified	17	10.3
Tenure	0–1 year	12	73.0
	<1–3 years	42	25.5
	<3–5 years	22	13.3
	<5–10 years	50	31.1
	<10–15 years	22	13.3
	<15 years	17	10.3

The blue-collar worker sample (*n* = 398) had a mean age of 35.78 years. The majority of the participants (85.4%, *n* = 340) were male, with 68.8% (*n* = 274) holding a high school diploma and 68.3% (*n* = 272) being single. A significant proportion (86.9%, *n* = 346) were engaged in shift work. Regarding work experience, 27.1% (*n* = 108) had been employed for 5–10 years, while 20.1% had worked for <15 years, and 18.1% had 10–15 years of experience. In terms of employment contracts, 71.9% (*n* = 286) held permanent contracts and were unionized, whereas 28.1% (*n* = 112) were employed under temporary contracts.

The white-collar worker sample (*n* = 165) had a mean age of 35.11 years. Among the participants, 58.2% (*n* = 96) were male, while 43.6% (*n* = 72) held a bachelor's degree, and 58.2% (*n* = 96) were single. A majority of white-collar workers (72.7%, *n* = 120) were not engaged in shift work. In terms of work experience, 31.1% (*n* = 50) had been employed for 3–5 years.

### 3.2 Measures

#### 3.2.1 Copenhagen psychosocial questionnaire (COPSOQ-III)

The psychosocial work environment was assessed using the third Turkish version of the Copenhagen Psychosocial Questionnaire (COPSOQ-III), a validated and reliable instrument designed to measure a broad spectrum of psychosocial risks within organizational settings. The Turkish adaptation and validation of COPSOQ-III, which comprises 78 items across 23 dimensions, was conducted by Sahan et al. (2019).

For the purposes of this study, three job demands—quantitative demand, cognitive demand, and emotional demand—were examined. The quantitative demand subscale consists of three items assessing the extent to which employees experience workload pressure (e.g., “*How often do you not have time to complete all your work tasks?”*). The cognitive demand subscale comprises four items, evaluating the extent to which the job requires sustained cognitive effort (e.g., “*Does your work require that you remember a lot of things?”*). The emotional demand subscale includes three items measuring the emotional burden placed on employees (e.g., “*Does your work put you in emotionally disturbing situations?”*). The internal consistency of these subscales, as measured by Cronbach's alpha, was 0.71, 0.66, and 0.78, respectively, indicating acceptable reliability levels.

Additionally, three key job resources—leader support, co-worker support, and trust—were included in the analysis. The leader support subscale consists of three items, assessing employees' perceived availability of managerial support (e.g., “*How often do you get help and support from your immediate superior, if needed?”*). The co-worker support subscale comprises three items, evaluating peer-based support mechanisms (e.g., “*How often do you get help and support from your colleagues, if needed?”*). The trust subscale includes four items, measuring employees' trust in managerial communication and decision-making (e.g., “*Can the employees trust the information that comes from the management?”*). The Cronbach's alpha values for these subscales were 0.84, 0.81, and 0.82, respectively, demonstrating strong internal reliability.

To examine work-related outcomes, this study focused on emotional exhaustion, job satisfaction, organizational commitment, and intention to leave. The emotional exhaustion subscale comprised four items, assessing the extent of fatigue resulting from work-related demands (e.g., “*How often have you felt tired?”*). The job satisfaction subscale included four items, evaluating overall contentment with one's job and future career prospects (e.g., “*Regarding your work in general, how pleased are you with your work prospects?”*). The organizational commitment subscale, specifically assessing commitment to the workplace, consisted of three items measuring the perceived significance of one's organization (e.g., “*Do you feel that your place of work is of great importance to you?”*). The Cronbach's alpha values for these subscales were 0.89, 0.84, and 0.76, respectively, indicating high internal consistency and reliability.

#### 3.2.2 Michigan organizational assessment questionnaire (MOAQ)

Employees' intention to leave their current positions was measured using the intention-to-leave subscale from the Michigan Organizational Assessment Questionnaire (MOAQ), originally developed by Cammann et al. (1979) and subsequently adapted into Turkish by Mimaroglu (2008). This subscale is designed to capture various dimensions of employees' turnover intentions. It comprises three items (e.g., “*I often think about quitting”*), rated on a five-point Likert scale ranging from 1 = “completely disagree” to 5 = “completely agree.” The subscale demonstrated acceptable internal consistency, with a Cronbach's alpha of 0.71, indicating sufficient reliability for measuring turnover intentions in the study sample.

### 3.3 Procedure

Data collection was conducted between October and December 2024. Participants were selected based on predefined inclusion criteria, which required them to be full-time employees holding either blue- or white-collar positions within the selected organizations. To facilitate data collection, different distribution methods were employed based on the occupational groups. White-collar workers received the questionnaire sets in their offices, where they were instructed to complete them individually and return them in a sealed box to ensure confidentiality. In contrast, blue-collar workers participated in the study during their designated break times, completing the questionnaires in a meeting room after receiving detailed instructions from the researchers. To further ensure ethical transparency, the study details and participation guidelines for blue-collar workers were communicated to the labor union they were affiliated with. Given that some participants, particularly blue-collar workers, had limited digital access at work, the study utilized paper-based questionnaires rather than online surveys to maximize accessibility and participation rates.

To uphold ethical integrity, all participants were provided with an information form detailing the study's objectives, confidentiality assurances, and their right to withdraw at any stage. Ethical approval was obtained from X University Scientific Research Ethics Committee, and all participants provided written informed consent prior to completing the questionnaire. Additionally, strict anonymity protocols were followed, ensuring that no personally identifiable information was collected, thereby maintaining participant confidentiality and data security.

### 3.4 Statistical analyses

All statistical analyses were conducted using Jamovi version 2.6.44 (The Jamovi Project, 2024). Descriptive statistics were computed to summarize the sociodemographic characteristics of the sample. Categorical variables were reported using frequencies and percentages, while continuous variables were described using means and standard deviations.

To test the hypothesized relationships among variables, multi-group structural equation modeling (MG-SEM) was applied to compare blue- and white-collar workers. MG-SEM allows for the simultaneous estimation of both measurement and structural components of the model, and enables an evaluation of whether the model functions equivalently across groups. The model was estimated using the maximum likelihood estimation method.

As a preliminary step, measurement invariance was tested to ensure that the constructs were measured equivalently across occupational groups. This process involved sequential testing of configural, metric, and scalar invariance models, following the recommendations of Vandenberg and Lance (2000). Configural invariance examined whether the overall factor structure was comparable across groups without imposing any equality constraints. Metric invariance tested whether factor loadings were equivalent, while scalar invariance assessed whether item intercepts were equal across groups. Invariance decisions were based on changes in model fit indices, with a ΔCFI ≤ 0.010 and a ΔRMSEA ≤ 0.015 indicating acceptable invariance (Chen, 2007; Cheung and Rensvold, 2002).

Following the assessment of measurement invariance, group differences in structural paths were examined to determine whether the relationships among latent variables varied across blue- and white-collar workers. Model fit was evaluated using multiple indices, including the chi-square to degrees of freedom ratio (χ^2^/df), Comparative Fit Index (CFI), Tucker–Lewis Index (TLI), Root Mean Square Error of Approximation (RMSEA), and Standardized Root Mean Square Residual (SRMR). Acceptable model fit was defined as χ^2^/df values between 2 and 3, CFI and TLI values of 0.90 or higher, and RMSEA and SRMR values between 0.05 and 0.08 (Kline, 2011).

To mitigate the risk of common method bias (CMB), several procedural and statistical precautions were implemented. First, participants were assured of anonymity and confidentiality, and scale items were distributed across different pages to reduce evaluation apprehension and response patterns. Second, different scale anchors and formats were used to psychologically separate constructs. Additionally, Harman's single-factor test was conducted to statistically assess CMB. The results showed that a single factor did not account for the majority of variance (first factor = 35%), indicating that common method bias was not a significant concern in this study (Podsakoff et al., 2003).

## 4 Results

### 4.1 Descriptive and bivariate correlational analyses of the main variables

Prior to conducting the main analysis, the dataset was examined for missing values and outliers. Cases with more than 10% missing data were excluded listwise (*n* = 12), and Little's MCAR test indicated that data were missing completely at random (χ^2^ = 86.12, *df* = 92, *p* = 0.63). For remaining cases, missing values were imputed using Expectation-Maximization (EM) estimation. Multivariate outliers were identified using Mahalanobis distance (*p* < 0.001), and five cases were removed due to excessive influence on model fit. These steps ensured the robustness and reliability of the final dataset used in MG-SEM analysis.

Table 3 presents the means, standard deviations, reliabilities, and correlations among the model variables. The results indicate that specific job demands, particularly quantitative and emotional demand, exhibit strong positive correlations with emotional exhaustion and intention to leave. Conversely, job resources, including leader support, co-worker support, and trust, are positively correlated with job satisfaction and organizational commitment. Additionally, these job resources demonstrate a significant negative correlation with intention to leave.

**Table 3:** Bivariate correlation coefficients of the study variables.

**Variables**	***M***	***SD***	**1**	**2**	**3**	**4**	**5**	**6**	**7**	**8**	**9**	**10**
1. Quantitative demand	2.75	0.85	–	0.21^**^	0.48^**^	−0.09^*^	−0.04	−0.22^**^	0.41^**^	−0.27^**^	−0.13^**^	0.17^**^
2. Cognitive demand	3.87	0.70		–	0.35^*^	0.19^**^	0.14^**^	0.02	0.03	0.04	0.20^**^	0.002
3. Emotional demand	3.14	1.10			–	−0.13^**^	−0.06	−0.29^**^	0.41^**^	−0.30^**^	−0.17^**^	0.27^**^
4. Leader support	3.16	1.05				–	0.49^**^	0.48^**^	−0.20^**^	0.36^**^	0.32^**^	−0.28^**^
5. Co-worker support	3.40	0.92					–	0.33^**^	−0.07	0.26^**^	0.26^**^	−0.19^**^
6. Trust	3.06	0.91						–	−0.32^**^	0.59^**^	0.44^**^	−0.45^**^
7. Emotional exhaustion	3.33	0.94							–	−0.39^**^	−0.26^**^	0.29^**^
8. Job satisfaction	2.71	0.70								–	0.52^**^	−0.59^**^
9.Organizational commitment	3.74	1.01									–	−0.49^**^
10. Intention to leave	2.37	1.14										–
	**Cronbach alpha**	0.71	0.66	0.78	0.84	0.81	0.82	0.89	0.84	0.76	0.82

* p <0.05,

** p <0.001.

### 4.2 Testing of the hypothesized model

#### 4.2.1 Measurement invariance testing across blue- and white-collar workers

To evaluate whether the measurement model operated equivalently across occupational groups, measurement invariance testing was conducted within the multi-group structural equation modeling (MG-SEM) framework. Configural, metric, and scalar invariance models were tested sequentially to examine the comparability of factor structures, factor loadings, and item intercepts across blue- and white-collar workers. Decisions regarding model invariance were based on changes in model fit indices, and a summary of the results is presented in Table 4.

**Table 4:** Measurement invariance tests across groups.

**Model**	***x^2^***	***df***	**CFI**	**RMSEA**	**SRMR**	**Comparison**	**ΔCFI**	**ΔRMSEA**
M0 (Configural)	1,971	1,026	0.898	0.057	0.088	–		
M1 (Metric)	2,030	1,054	0.895	0.057	0.093	M0 vs. M1	0.003	0.000
M2 (Scalar)	2,162	1,076	0.883	0.06	0.094	M1 vs. M2	0.012	0.003

The configural model, in which no equality constraints were imposed, demonstrated acceptable fit to the data (χ^2^ = 1,971, *df* = 1,026, CFI = 0.898, RMSEA = 0.057, SRMR = 0.088), suggesting that the overall factor structure was consistent across groups.

The metric invariance model, in which factor loadings were constrained to be equal across groups, also showed acceptable model fit (χ^2^ = 2,030, *df* = 1,054, CFI = 0.895, RMSEA = 0.057). The differences in model fit between the configural and metric models (ΔCFI = 0.003; ΔRMSEA = 0.000) were within acceptable thresholds, supporting metric invariance.

In the scalar invariance model, both factor loadings and item intercepts were constrained to be equal. Although model fit slightly declined (χ^2^ = 2,162, *df* = 1,076, CFI = 0.883, RMSEA = 0.060), the observed changes in fit (ΔCFI = 0.012; ΔRMSEA = 0.003) were minimal. Given that the change in RMSEA remained within acceptable limits and the ΔCFI only marginally exceeded the 0.010 criterion, partial scalar invariance was deemed acceptable, in line with the recommendations of Byrne et al. (1989).

#### 4.2.2 Structural path analysis

**Figure 2:**
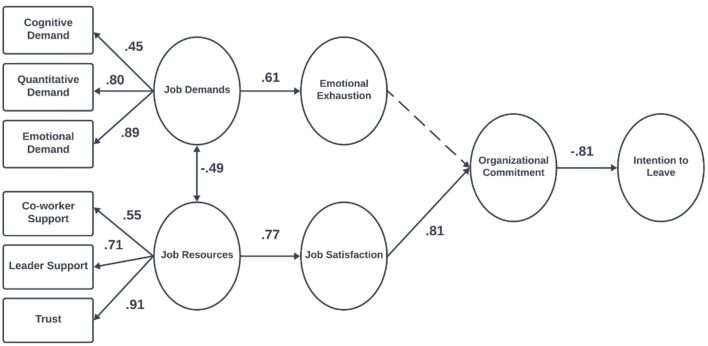
Maximum likelihood estimates of the JD-R theory for blue-collar workers, *N* = 398. The numbers in the figure represent standardized regression coefficients, *p* < 0.001.

**Figure 3:**
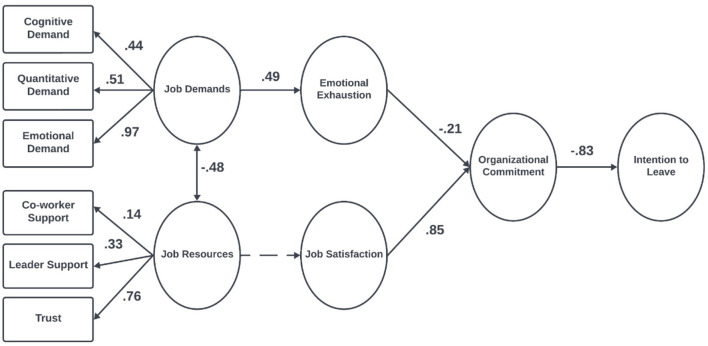
Maximum likelihood estimates of the JD-R theory for white-collar workers, *N* = 145. The numbers in the figure represent standardized regression coefficients, *p* < 0.001.

Given that measurement invariance was established, the hypothesized structural model was estimated separately for the blue-collar and white-collar groups without imposing equality constraints. This approach enabled the examination of potential group-specific differences in path coefficients and mediation mechanisms. All indicators exhibited strong factor loadings on their respective constructs (see Figures 2, 3). The unstandardized (b) and standardized (β) coefficients from the group-specific models are presented in Tables 5, 6.

**Table 5:** Parameter estimates of the direct paths in the model.

**Worker type**	**Structural paths**	***b***	**95% Confidence intervals**		β	***p***
			**Lower**	**Upper**		
**Direct paths**						
Blue-collar workers	Emotional exhaustion Demands ⇒ Emotional exhaustion	0.864	0.646	1.083	0.61	**<0.001**
	Job satisfaction Resources ⇒ Job satisfaction	1.235	1.003	1.692	0.774	**<0.001**
	Organizational commitment EE ⇒ Organizational commitment Job satisfaction ⇒ OC	0.005 0.832	−0.068 0.646	0.080 1.020	0.007 0.815	0.875 **<0.001**
	Intention to leave Organizational commitment ⇒ ItL	−1.124	−1.376	−0.872	−0.81	**<0.001**
White-collar workers	Emotional exhaustion Demands ⇒ Emotional exhaustion	1.200	0.580	1.815	0.49	**<0.001**
	Job satisfaction Resources ⇒ Job satisfaction	7.122	−2.843	17.088	0.949	0.16
	Organizational commitment EE ⇒ Organizational commitment Job satisfaction ⇒ OC	−0.101 0.470	−0.178 0.232	−0.023 0.701	−0.216 0.851	**0.010** **<0.001**
	Intention to leave Organizational commitment – ItL	−2.186	−3.289	−1.090	−0.831	**<0.001**

EE, Emotional Exhaustion; OC, Organizational Commitment; ItL, Intention to Leave; JS, Job Satisfaction. The bold values are used to visually indicate statistically significant *p*-values (*p* < 0.05) and facilitate interpretation.

**Table 6:** Parameter estimates of the indirect paths in the model.

**Worker type**	**Structural paths**	***b***	**95% confidence intervals**		β	***p***
			**Lower**	**Upper**		
**Indirect paths**						
Blue-collar workers	Demand ⇒ EE ⇒ OC ⇒ ItL	−0.006	−0.078	0.067	−0.004	0.88
	Resources ⇒ JS ⇒ OC ⇒ ItL	−1.262	−1.625	−0.899	−0.511	**<0.001**
	EE ⇒ OC ⇒ ItL	−0.007	−0.090	0.077	−0.006	0.88
	JS ⇒ OC ⇒ ItL	−0.936	−1.107	−0.766	−0.660	**<0.001**
White-collar workers	Demand ⇒ EE ⇒ OC ⇒ ItL	0.264	0.056	0.472	0.088	**0.01**
	Resources ⇒ JS ⇒ OC ⇒ ItL	−7.325	−17.62	0.279	−0.67	0.163
	EE ⇒ OC ⇒ ItL	0.220	0.085	0.356	0.180	**0.001**
	JS ⇒ OC ⇒ ItL	−1.029	−1.258	−0.779	−0.710	**<0.001**

EE, Emotional Exhaustion; OC, Organizational Commitment; ItL, Intention to Leave; JS, Job Satisfaction. The bold values are used to visually indicate statistically significant *p*-values (*p* < 0.05) and facilitate interpretation.

In both blue-collar (β = 0.61, *p* < 0.001) and white-collar (β = 0.49, *p* < 0.001) workers, job demands were directly associated with emotional exhaustion. Additionally, a direct relationship between job resources and job satisfaction was observed among blue-collar workers (β = 0.77, *p* < 0.001), whereas this relationship was not significant for white-collar workers (β = 0.95, *p* = 0.16).

Job satisfaction was positively associated with organizational commitment in both blue-collar (β = 0.82, *p* < 0.001) and white-collar workers (β = 0.85, *p* < 0.001). However, emotional exhaustion was found to be directly related to organizational commitment only among white-collar workers (β = −0.21, *p* = 0.01). Finally, in both blue-collar (β = −0.81, *p* < 0.001) and white-collar workers (β = −0.83, *p* < 0.001), organizational commitment was negatively associated with intention to leave.

Among blue-collar workers, job satisfaction and organizational commitment sequentially mediated the relationship between job resources and intention to leave (β = −0.51, *p* < 0.001). Additionally, organizational commitment alone served as a mediator between job resources and intention to leave (β = −0.66, *p* < 0.001). However, emotional exhaustion and organizational commitment did not sequentially mediate the relationship between job demands and intention to leave (*p* = 0.88). Furthermore, organizational commitment did not mediate the indirect pathway from emotional exhaustion to intention to leave (*p* = 0.88) among blue-collar workers.

Among white-collar workers, emotional exhaustion and organizational commitment sequentially mediated the relationship between job demands and intention to leave (β = 0.09, *p* = 0.01). Additionally, organizational commitment mediated the relationship between emotional exhaustion and intention to leave (β = 0.18, *p* = 0.001), as well as job satisfaction and intention to leave (β = −0.71, *p* < 0.001). However, job satisfaction and organizational commitment did not sequentially mediate the relationship between job resources and intention to leave (*p* = 0.16).

## 5 Discussion

This study investigated the relationship between job demands, job resources, and work-related outcomes among both blue- and white-collar workers in Türkiye. The results of multi-group SEM revealed notable differences between these occupational groups, underscoring the importance of considering job-specific distinctions in workplace dynamics. These findings contribute to the growing body of research that emphasizes the differentiated functioning of the JD-R model across occupational strata (Bakker and Demerouti, 2017; Schaufeli and Taris, 2014). In particular, they highlight how the relative weight of motivational vs. health-impairment processes may vary depending on structural factors such as task complexity, autonomy, support systems, and labor protections. By incorporating occupational segmentation into its design, the study responds to calls for greater contextualization of work-related stress models, especially in non-Western, industrialized settings (Koseoglu et al., 2021; Tomczak and Kulikowski, 2023).

The support for H_1_ confirms the central premise of the JD-R theory's health impairment process, which argues that prolonged exposure to job demands depletes individuals' psychological and emotional resources, resulting in emotional exhaustion. This mechanism is particularly salient when multiple demands—such as emotional, cognitive, and quantitative—are experienced simultaneously; leading to a compounding effect that heightens the risk of strain beyond what each demand might produce individually. The cumulative burden of these demands reflects the interactive stressor amplification process, which has been recognized in prior research emphasizing multi-source role strain (Bakker and Demerouti, 2006; Jimmieson et al., 2017). The present finding is also consistent with the work of Demiral et al. (2023), who identified significant associations between job demands and poor mental health outcomes among Turkish employees across occupational groups. Similar relationships have been documented in Western settings, where quantitative workload and emotional labor consistently predict exhaustion (Lee and Ashforth, 1996; Schaufeli and Taris, 2014). However, while prior studies often examine these demands independently, our findings highlight the importance of considering their co-occurrence. No contradictory evidence was found regarding this relationship in our dataset; emotional exhaustion appears robustly driven by aggregated demands in both blue- and white-collar contexts, without significant divergence from the established JD-R assumptions.

The partial support for H_2_ reveals that job resources positively influence job satisfaction only among blue-collar workers, pointing to occupational divergence in how social support mechanisms are internalized. Theoretically, this aligns with need-based motivational frameworks suggesting that workers prioritize different types of resources depending on their role characteristics and socio-economic positioning. For blue-collar employees, who often have limited autonomy and repetitive tasks, interpersonal support may serve as a critical external affirmation of value and stability, satisfying their need for relatedness and security (Deci and Ryan, 2000; Lin-Hi et al., 2022). The strong association between job satisfaction and leader or co-worker support in this group is consistent with prior research indicating that relational dynamics are especially salient in low-control environments (Lips-Wiersma et al., 2016; Saari et al., 2022). In contrast, the absence of a significant relationship among white-collar workers may stem from a mismatch between the type of resources examined and the motivational structures that guide their satisfaction. White-collar roles often involve higher levels of cognitive demand, autonomy, and growth expectations; thus, these employees may derive satisfaction from intrinsic or developmental resources such as role clarity, autonomy, or skill use—dimensions not directly captured in the present study (Chung et al., 2010; Wheatley, 2017). This interpretive gap may explain the absence of significance in this subgroup, rather than an outright contradiction of the JD-R theory.

The finding that organizational commitment mediates the relationship between job satisfaction and intention to leave across both occupational groups (H_3_) underscores the centrality of affective attachment in turnover decisions, as posited by commitment-based models of employee retention (Meyer et al., 1993; Allen and Meyer, 1996). This result is theoretically consistent with the JD-R theory's motivational pathway, in which job satisfaction fosters a sense of loyalty and reduces the likelihood of voluntary departure. It is also empirically supported by prior research across sectors and cultures (Guzeller and Celiker, 2020; Samad and Yusuf, 2012), confirming that organizational commitment remains a robust and stable mediator regardless of occupational role. However, the partial support for H_4_–where commitment mediated the emotional exhaustion–turnover link only for white-collar workers—suggests that the health impairment mechanism operates asymmetrically. One plausible interpretation is that white-collar employees, who often have greater autonomy and career visibility, are more likely to translate psychological fatigue into exit intentions when organizational commitment weakens (Lee and Ashforth, 1996; Sverke et al., 2002). In contrast, blue-collar workers may experience high levels of exhaustion but are less inclined or less able to act on it due to limited external opportunities, economic dependency, or unionized job protections. This occupational constraint may suppress the mediating role of commitment in the exhaustion–turnover pathway, not because the emotional toll is absent, but because it does not manifest behaviorally in the same way.

The partial support for H5—indicating that emotional exhaustion and organizational commitment sequentially mediate the relationship between job demands and intention to leave, but only among white-collar workers—adds further nuance to the JD-R theory's health impairment pathway. Specifically, heightened job demands in white-collar roles trigger emotional exhaustion, which subsequently erodes organizational commitment and increases turnover intentions. This process is well-documented in the literature, with studies across sectors, such as nursing and hospitality, confirming that burnout mediates the link between job demands and withdrawal behaviors (Babakus et al., 2008; Jourdain and Chênevert, 2010; Rahnfeld et al., 2023). However, the occupational specificity observed in this study suggests that the activation of this pathway is not solely psychological but also structurally determined. In Türkiye, white-collar workers are particularly vulnerable to cumulative role overload due to blurred boundaries between work and personal life, widespread unpaid overtime, and implicit expectations of after-hours availability (Anxo and Karlsson, 2019). These conditions amplify cognitive and emotional demands and intensify chronic stress, undermining affective ties to the organization. Over time, emotional exhaustion in such roles translates more readily into turnover intentions, particularly when organizational commitment declines. In contrast, blue-collar workers typically operate under more regulated shift structures with clearer role boundaries and compensation protocols, which may buffer the psychological consequences of job demands. Their greater job predictability and work-life separation reduce the likelihood that emotional strain will translate into active exit considerations, even when exhaustion is present. Therefore, the absence of a significant mediation effect in this group does not imply a lack of emotional burden but reflects a constrained behavioral response shaped by structural, contractual, and socioeconomic limitations. These findings highlight that the health impairment process in the JD-R theory, while theoretically generalizable, manifests unevenly across occupational strata, reinforcing the need for stratified intervention strategies in retention management.

Continuing this line of analysis, the partial support for H_6_–where the sequential pathway from job resources to turnover intentions via job satisfaction and organizational commitment was significant only among blue-collar workers—illustrates how the JD-R theory's motivational process operates unevenly across occupational roles. Theoretically, this aligns with the notion that job resources stimulate motivation by fulfilling basic psychological needs (Deci and Ryan, 2000), thereby increasing satisfaction and commitment and reducing the likelihood of exit (Bakker and Demerouti, 2017). In blue-collar contexts, where task autonomy and developmental opportunities are typically limited, interpersonal resources such as leader and coworker support become particularly salient in shaping positive work attitudes (Ersoy et al., 2011; Schreurs et al., 2011). Prior studies have found that blue-collar workers are more dependent on relational support to establish trust and engagement, which in turn solidifies organizational commitment (Ito and Brotheridge, 2005; Russell et al., 2020). Our findings resonate with these patterns, especially in collectivist and high power distance cultures like Türkiye, where supportive hierarchical relationships are critical for fostering retention. The absence of this mediation pathway among white-collar workers may be attributed to their preference for intrinsic or cognitive resources—such as autonomy or challenge—over relational support. Consequently, the social job resources measured in this study may have been insufficient to activate the same satisfaction–commitment–retention mechanism in that group, pointing not to a theoretical inconsistency but to a mismatch between resource type and motivational drivers.

Finally, the confirmation of H_7_ provides empirical support for the occupational stratification of JD-R processes, revealing that the relative weight of job demands and job resources on work-related outcomes differs significantly between blue- and white-collar workers. Theoretically, this reinforces the dual-pathway assumption of the JD-R theory, but nuances it by showing that the motivational process (resources → satisfaction → commitment → retention) is more salient among blue-collar workers, whereas the health impairment process (demands → exhaustion → commitment erosion → turnover) predominates among white-collar workers. This bifurcation supports prior theoretical suggestions that the dominance of these pathways may be contingent on contextual and occupational variables (Bakker and Demerouti, 2017; Korunka et al., 2009). For blue-collar employees, whose work tends to be structured, repetitive, and externally controlled, the availability of relational job resources—particularly trust and coworker support—acts as a vital stabilizing factor, as evidenced in prior research from unionized settings (Böckerman et al., 2012; Schreurs et al., 2011). In contrast, white-collar workers operate within more individualized employment arrangements and often value autonomy, intellectual challenge, and task significance over interpersonal support (Weaver, 1975). This may explain why job resources failed to buffer against turnover intentions in this group, while job demands had a direct and more damaging effect. Rather than conflicting with the JD-R theory, these findings illustrate how the salience of each pathway is shaped by role expectations, employment contracts, and professional norms. The results also highlight the need for a differentiated application of JD-R constructs across occupational classes, especially in socioeconomically diverse labor markets like Türkiye.

### 5.1 Limitations, strengths, and implications

This study has several limitations that should be acknowledged. First, its cross-sectional design restricts the ability to draw causal inferences, as the observed relationships represent correlational patterns measured at a single point in time. Such limitations are common in JD-R research, where causal ordering is inferred theoretically but cannot be empirically established without longitudinal data (Taris and Kompier, 2003). Second, the sample exhibits a gender imbalance (106 women vs. 436 men), which, while reflective of Türkiye's industrial sector composition, may constrain the generalizability of the findings—particularly with respect to female workers. According to TurkStat (2024), men comprise the majority of the industrial workforce (~4,979,000 men vs. 1,751,000 women), and our sample mirrors this structural imbalance. Nevertheless, previous studies have shown that men and women can differ in how they experience and respond to job demands and resources (Llorens et al., 2006; Purvanova and Muros, 2010). For example, emotional exhaustion, perceptions of fairness, and supervisor support may vary by gender. Therefore, interpretations of gendered work experiences should be approached with caution. Future studies should aim for more gender-balanced sampling or investigate gender as a potential moderating variable to explore these nuances more systematically.

A third limitation concerns the potential for self-selection bias arising from the voluntary nature of participation. Employees who chose to take part in the study may systematically differ from those who did not, particularly in terms of engagement or dissatisfaction. For instance, individuals with stronger opinions—whether positive or negative—may have been more inclined to respond, which could influence the variability and representativeness of the findings. Although our sample is broadly aligned with the occupational and demographic characteristics of the sector, this form of bias should be considered when interpreting the generalizability of the results. Future research may benefit from using randomized sampling procedures or including measures of participation motivation to assess and address self-selection effects.

Despite these limitations, this study makes two notable contributions to the JD-R literature. First, it extends existing research by empirically examining the impact of job demands and resources on work-related outcomes in a non-Western, culturally distinct context. To our knowledge, it is the first study in Türkiye to test the sequential effects of job demands and resources on turnover intentions, addressing a gap identified by Koseoglu et al. (2021) in their systematic review. Second, by differentiating between blue- and white-collar workers, the study reveals occupationally distinct mediation pathways, highlighting how the JD-R theory functions differently across workforce segments. Methodologically, the application of multi-group structural equation modeling (MG-SEM) strengthens the robustness of cross-occupational comparisons and offers empirical validation for the JD-R theory's dual-process structure.

The findings also carry practical implications for human resources (HR) management and policy development. For blue-collar workers, strengthening interpersonal job resources—such as supervisor support, co-worker relationships, and organizational trust—can effectively reduce turnover intentions by promoting job satisfaction and enhancing organizational commitment. These findings support inclusive HR strategies such as leadership development, peer mentoring, and relational trust-building. For white-collar workers, the results emphasize the importance of addressing emotional exhaustion through demand-management strategies. These may include mental health support programs, flexible work policies, and clearer delineation of working hours to alleviate role overload and foster work-life balance. These interventions may be especially effective in high power distance and collectivist cultures like Türkiye, where employees place strong value on interpersonal trust and hierarchical support (Aycan, 2006; Hofstede, 2001).

One notable finding warrants further theoretical consideration. While job resources significantly predicted job satisfaction among blue-collar workers, this relationship was not observed among white-collar workers. This divergence may reflect occupational differences in motivational orientation. Based on self-determination theory (Deci and Ryan, 2000), white-collar workers may prioritize intrinsic factors such as autonomy, skill utilization, and growth opportunities—elements not directly captured by the relational resources measured in this study. Previous research supports this interpretation, showing that employees in higher-status roles often derive satisfaction from developmental and task-related resources rather than interpersonal support (Giauque et al., 2016; Kuvaas et al., 2017). These differences suggest that resource-based interventions should be tailored to the value structures and occupational identities of different worker groups. Future studies should explore whether resource–satisfaction pathways differ by occupational status, particularly in hybrid or remote work settings where conventional support structures may be less salient.

Lastly, this research aligns with key Sustainable Development Goals (SDGs), notably SDG 3 (Good Health and Wellbeing), SDG 8 (Decent Work and Economic Growth), and SDG 10 (Reduced Inequalities). By identifying how occupational roles influence the relationship between working conditions and mental wellbeing, the study provides an evidence base for labor policies aimed at improving psychosocial work environments—particularly for vulnerable or underrepresented employee groups in developing economies. This aligns with calls for integrating occupational health and psychosocial risk management into the global sustainability agenda (Leka and Nicholson, 2019).

## Data Availability

The original contributions presented in the study are included in the article/supplementary material, further inquiries can be directed to the corresponding author.
